# RoseVisuals: A Multi-Class Dutch Rose Petal Images Dataset for Automated Health and Pigmentation Classification via Deep Learning

**DOI:** 10.1038/s41597-026-07630-4

**Published:** 2026-06-23

**Authors:** Arya Sabale, Devashri Kapse, Mahek Mehta, Rutuja Rajendra Patil, Gagandeep Kaur, Ghanshyam G. Tejani, Harshada Vishal Mhetre, Anuradha Yenkikar, Amol V. Patil, A. S. Veerendra

**Affiliations:** 1 Department of Computer Science Engineering – Artificial Intelligence and Machine Learning, Vishwakarma Institute of Information Technology, Pune, India; 2 Department of Computer Engineering, MIT, Academy of Engineering, Alandi, Pune, India; 3 Department of Computer Engineering, Mukesh Patel School of Technology, Management and Engineering, SVKM’s NMIMS, Shirpur, India; 4 Department of Research Analytics, Saveetha Dental College and Hospitals, Saveetha Institute of Medical and Technical Sciences, Saveetha University, Chennai, 600077 India; 5 Department of Industrial Engineering and Management, Yuan Ze University, Taoyuan, 320315 Taiwan; 6 Department of Electronics & Communication Engineering, Bharati Vidyapeeth (Deemed University) College of Engineering, Pune, India; 7 Department of Computer Science Engineering – Artificial Intelligence, Vishwakarma Institute of Technology, Pune, India; 8 Department of Computer Science Engineering-Artificial Intelligence, Vishwakarma Institute of Technology, Pune, India; 9 Manipal Institute of Technology, Manipal Academy of Higher Education, Manipal, India

**Keywords:** Agriculture, Forestry

## Abstract

The robust Dutch rose, also known as the *Rosa hybrida* is distinguished by its vibrant colors, superior product quality, and extended vase life. These rose varieties, originating from Netherlands, have proven highly successful in Indian agricultural conditions and the international export industry. The dataset consists of a total of 1,995 high resolution petal image collected during this research, encompassing petal color categories, such as red, yellow, white, pink, purple, orange, bi-color, and multi-color, as well as health statuses including fresh, dry, and diseased petals. The primary purpose of this dataset is to support machine learning activities in agriculture and specifically for tasks such as automatic petal health evaluation and rose variety categorization. Although the rose flower is scientifically rich and has a wide range of industrial uses, it has not been given much attention in machine learning, especially when compared to other plant-based datasets. This study adds to the accuracy of quality assessment through the use of modern computer vision and machine learning methods, thus helping the agriculture sector, rose-based edible product making, and flavor development industries.

## Background & Summary

The floriculture industry regards the Dutch rose varieties as an essential element because of their beneficial characteristics that are not only of great commercial value but also have various industrial applications besides merely being collectibles. These roses, which are especially famous in Talegaon-Maval and Marketyard near Pune, serve decorative, medicinal, flavour development, and industrial purposes. Dutch rose petals serve dual purposes: they enhance the taste and visual appeal of food and beverages, and are also widely used in cosmetics, oil extraction for perfumes, dyeing, also as a crafting material in textiles. These versatile applications have given Dutch Roses significant economic value across multiple sectors. Despite being profitable commercially, Dutch Rose presents various challenges. This includes suffering from specific diseases and post-harvest issues that can degrade petal quality, affecting market value Although several floral datasets exist for species recognition and leaf-based disease detection, publicly available datasets dedicated to petal-level rose analysis that jointly capture color diversity and petal health conditions remain extremely limited. Existing datasets largely focus on whole flowers or leaves, which restricts fine-grained assessment of petal-specific morphological and pigmentation changes^1^. Full-plant images give a general idea, but rarely with the detail and resolution that enables early-stage stress or disease symptoms to be identified, particularly in flowering species such as roses. In contrast, petal-level visual analysis provides fine-grained features such as fading, edge curl, drying, fungal spots, and morphological changes, which are the key (invisible) indicators that do not show in the wide-angle or leaf-based images. This study examines Dutch rose petals, which are distinguished by their unique features. Building accurate machine-learning models depends upon the creation of tailored datasets that capture these distinct traits^2^. The proposed dataset also includes images of diseased petals, which are valuable in building precise machine-learning models for plant disease detection^3^. Such tools have the potential to improve disease detection and enable smarter quality control, thereby helping industries that depend on these rose extracts.

### Information

**Fig. 1:**
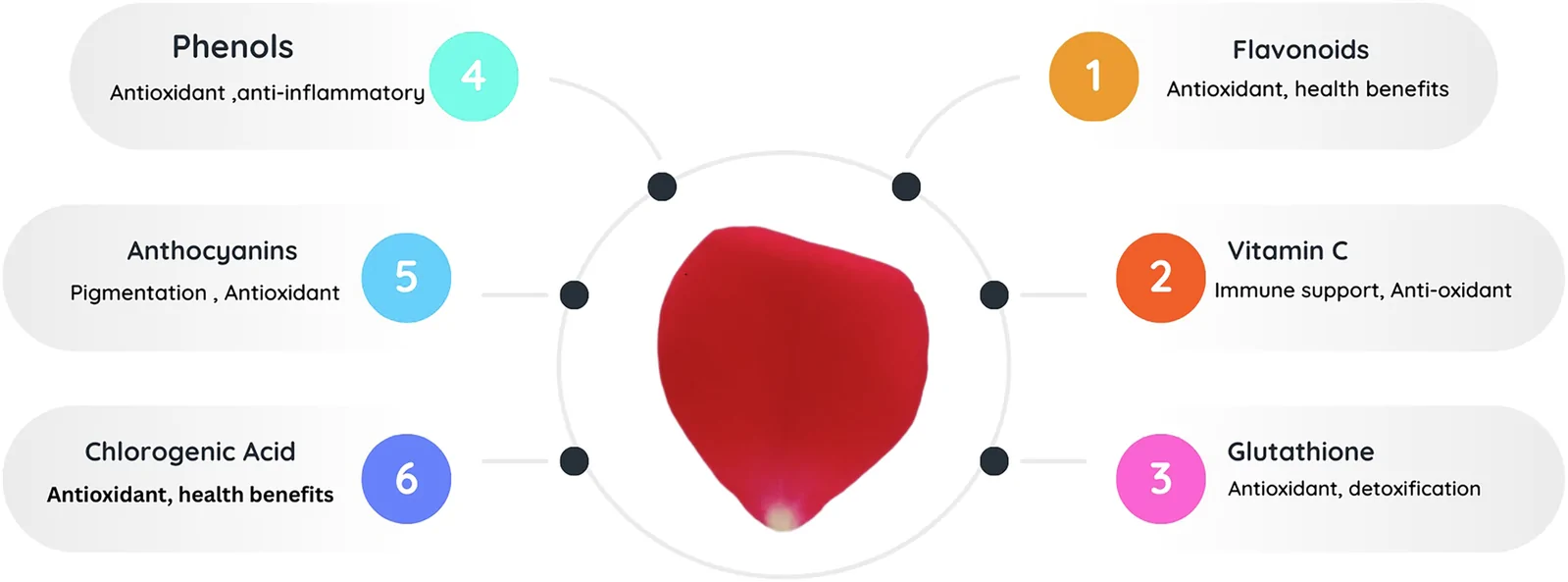
Reported bioactive compounds and associated functional properties of Dutch rose petals based on literature.

Advanced greenhouse agricultural techniques in both the Netherlands and India help the efficient development of bioactive compounds found in Dutch rose petals. Wild roses (genus Rosa) typically produce five petals, while Dutch varieties produce multiple-layered petals, making them distinct from other rose types. Anthocyanin pigments, which are responsible for flower coloration, also influence petal health due to their biochemical properties^4^. Chlorogenic acid is found in all petals. Dutch roses are known to contain vitamin C, glutathione, and various antioxidants^5^. These compounds are commonly reported in literature to be associated with antioxidant and pigmentation related properties in plant tissues. As illustrated in Fig. 1, Dutch rose petals are reported in literature to contain flavonoids, anthocyanins, phenols, chlorogenic acid, vitamin C, and glutathione.

Applications

- The proposed diverse dataset consists of various color and health categories of Dutch rose petal images. This wide range of botanical materials serves two main purposes: inventory management in floriculture and quality assessment or selection of inputs for the cosmetics, food, and perfumery industries.
- The dataset can thus assist researchers in proposing innovative algorithms for the analysis of the texture of petals, color patterns, and shape variations, especially in application-based studies of diseases and rose varieties within computer vision and pattern recognition.
- Food scientists and culinary researchers develop automated petal quality assessment systems using high-quality images of fresh petals. The methodology ensures consistency and reliability in outcomes when dealing with rose petals in food preparation.
- The cosmetics and natural products industry employs formulators and researchers to inspect the visual characteristics of the dataset to explore the correlation between color traits and natural compounds.
- The outcomes of this study facilitate the practice of systematically evaluating rose varieties and picking those suited for medicinal use. Exploratory visual analysis for selecting rose petals for traditional and functional product research. The dataset is not equipped with biochemical or clinical measurements and is not aimed at direct medical diagnosis.

## Methods

### Experimental design

**Fig. 2:**
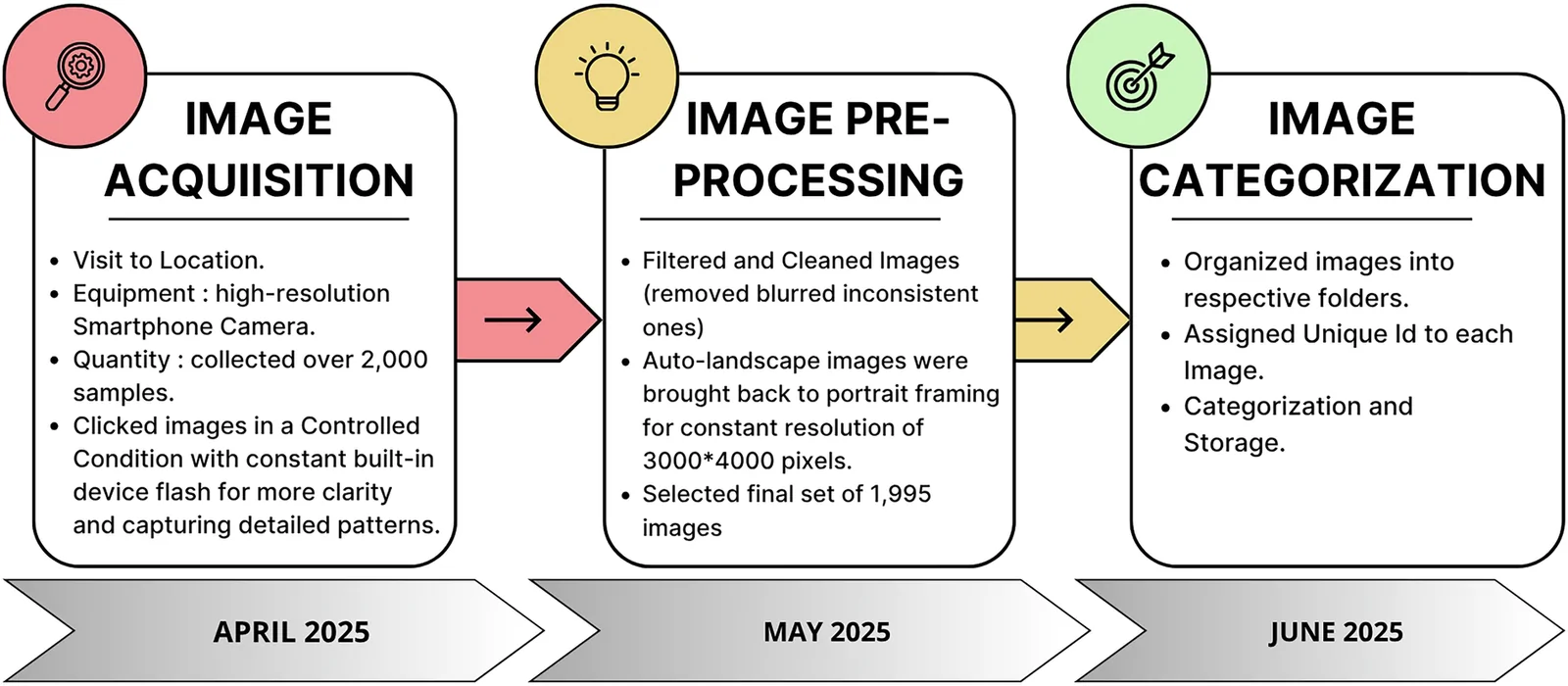
Experimental Workflow.

A structured and process was followed throughout the creation of the RoseVisuals dataset right from sample collection to final image labelling. The petals were directly collected from farms but petal images were captured under controlled environment with stable location and built in flash, whereas all the pictures are taken in the same standard set up with standardized lighting, background and uniform camera settings to guarantee consistency in imaging. Figure 2 depicts the end-to-end workflow used to prepare and validate the dataset.

### Phase 1: Image acquisition

Peatls samples were collected from commercial Dutch rose farms located in Talegaon-Maval (18.7350 °N, 73.6756 °E) and the Pune Market Yard region (18.5018 °N, 73.8636 °E), Maharashtra, India. All petal images were taken in controlled situations to guarantee. the fact that they are suitable to be used commercially. A Samsung Galaxy S21 Ultra (Smart Model SM-G998B). The smartphone was used with a 7 mm focal length lens (equivalent of 35 mm: 24 mm), to take the shot. Built in flash of Samsung Galaxy S21 Ultra was used to eradicate shadow and keep true colors which is essential to the evaluation of natural dyes. All pictures were taken on white background. The camera settings were maintained consistent during the process of taking the image, the aperture was f/1.8, the shutter speed was 1/771 s, and ISO set to 80. The images were taken in the 10:00 AM 2:00 PM local time to reduce changes in ambient lighting conditions under consistent flash light. Ambient temperature when data was collected was between 25–31 °C in clear weather. All the images were taken in the house or shaded arrangements, and photos were taken right after collecting the petals to prevent interference by the sunlight. The camera to petal distance was kept constant at about 15 cm during the entire process of acquiring. The phone was handheld and provide a steady framing. The device automatically controlled the white balance, and kept it constant on all samples, whereas the in-built flash was turned on so that the illumination and color distortion by changes in ambient light were standardized.

All images were saved in JPG format with a consistent resolution of 3000 × 4000 pixels with minimal compression to maintain visual reliability.

### Phase 2: Image Pre-processing

After image acquisition, the team proceeded to process each image that was captured carefully in order to get to the desired result. During preprocessing, blurred and out-of-focus images that demonstrated uneven lighting were manually removed in order to preserve the quality of the dataset. Some of the images were originally captured in auto landscape mode, were uniformly rotated to portrait mode for consistent framing without altering any of the properties of the image. This orientation correction has been done without any duplication or use of static manual augmentation techniques. Dry petals were produced through open-air sun drying under natural environmental conditions, with daily exposure of approximately 5–6 hours at ambient temperatures ranging from 25–31 °C. No controlled drying chamber was used. The original resolution of 3000 × 4000 pixels was preserved throughout the entire pre-processing methodology.

### Phase 3: Image categorization & organization

The whole dataset was sorted firstly based on petal health and then sub categorized based on visually visible colors into 8 different categories. A systematic naming convention (unique identification number) was given to the final set of images, combined with petal status descriptions and number sequences that represented color classifications. The classification of petal color was done visually using the perception of a supreme hue as opposed to a standardized colorimetric reference like the RHS Colour Chart. The color of the individual petals was given as a category (red, yellow, white, pink, purple, orange, bi-color and multi-color) according to the visual domination pigmentation when exposed to standardized lighting conditions. The two or more different pigment regions could be identified on one petal and therefore the bi-color and multi-color categories were used. This practical categorical methodology mirrors the industrial sorting methods that are applied in the floriculture industry and post-harvest industries. Diseased petals were identified based on clearly visible surface abnormalities, including fungal spots, necrotic regions, discoloration, and tissue breakdown. While symptoms consistent with common rose diseases such as black spot, rust, and botrytis were observed, the dataset does not assign disease-specific diagnostic labels, as no laboratory or microscopic confirmation was conducted. Images were therefore conservatively labeled label led as “diseased” only when visual symptoms were unambiguous, reducing the risk of misclassification. For Example, FRESH_RED_001 refers to the first sample of fresh red petals. In the naming format, FRESH indicates the health condition of the petals, and RED specifies the color variety, and 001 denotes the individual sample number. Table 1 provides the sample images from the RoseVisuals dataset.

**Table 1:** Sample Images.

Petal Health Condition	Sample Images of Petals Classified according to Color category
Fresh Petals Sample Images	
Dry Petals Sample Images	
Diseased Petals Sample Images	

To show the Sample images of the dataset RoseVisuals with the instances categorized based on petal health and colors present. Each row displays the sample examples of various color categories such as Bi-color, Multi-color, Orange, Pink, Purple, Red, White, and Yellow folders of the RoseVisuals Dataset. The colors and textural integrity of the products in the categories of Fresh and Dry are very diverse. The Diseased category points out diseases like discoloration, fungal development, and degradation, which are critical for training models to assess plant health.

### WorkFlow

**Fig. 3:**
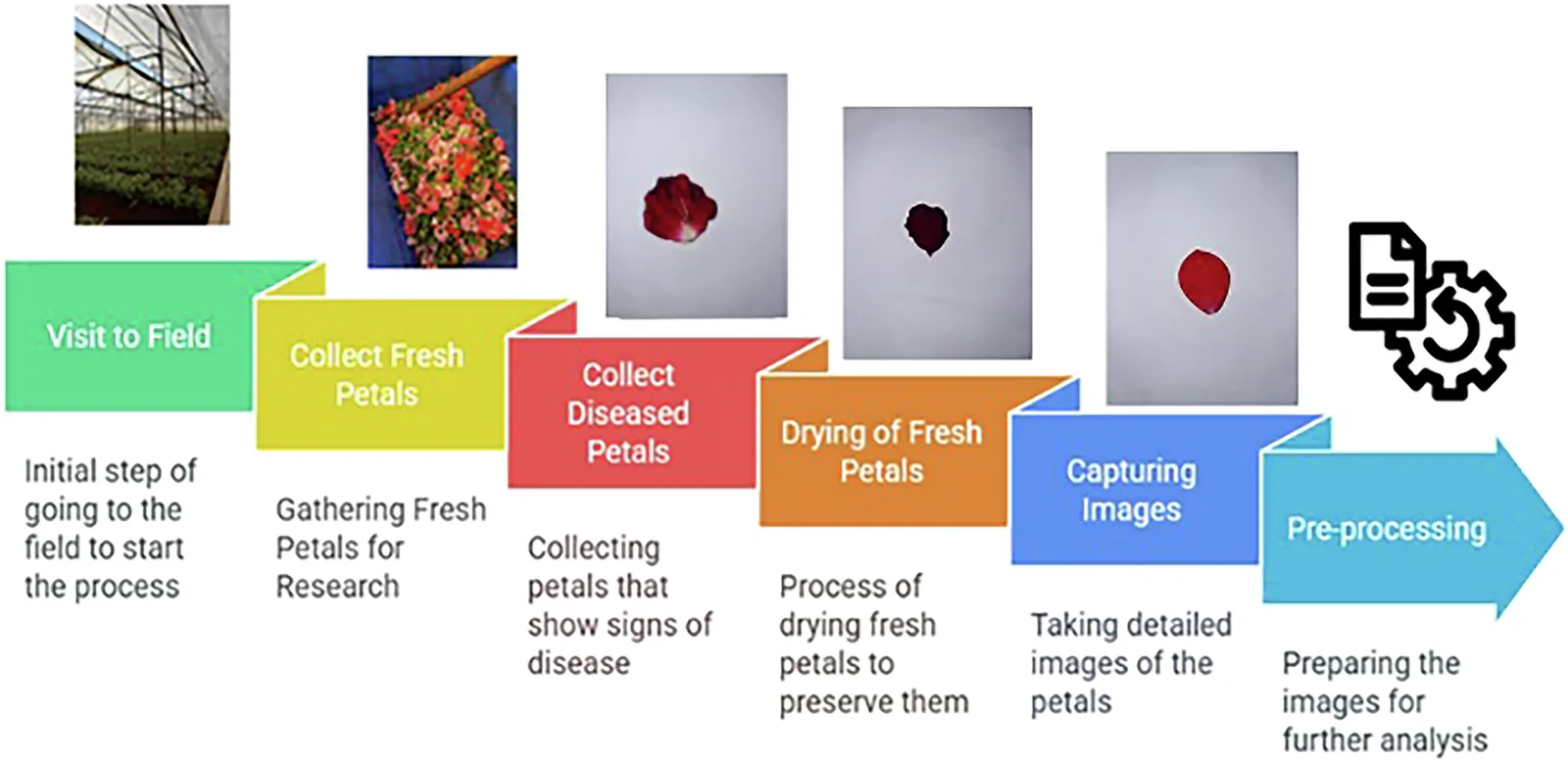
Research Workflow.

The dataset was developed on the basis of the samples, which were taken in the commercial rose farms in Talegaon-Maval, Pune, a region with a great diversity of rose plants and trade, with both healthy and diseased specimen. In case of dry samples, the drying experiment entailed the exposure of freshly collected petals to direct sunlight over a period of three days after which end pictures of the dried petals were obtained. All images of petals were captured at a given camera position having a white background to provide the visual accuracy and consistency. A detailed quality control was implemented in order to ensure the authentication and uniqueness of every image. The format and classification scheme of the RoseVisuals data set had been affected by standard guideline data sets such as the PriBeL dataset on betel leaf classification, which used standards such as fresh, infected, and dried leaves^6^. The study was carried out in a systematic manner starting with the selection of the site and sampling methodology, and processing of images. The image is of one intact petal, the petals are not overlapping according to the image so that the surface features of the petal remain intact. Figure 3 shows the detailed process followed to create the entire dataset.

## Data Records

### Data organization

**Fig. 4:**
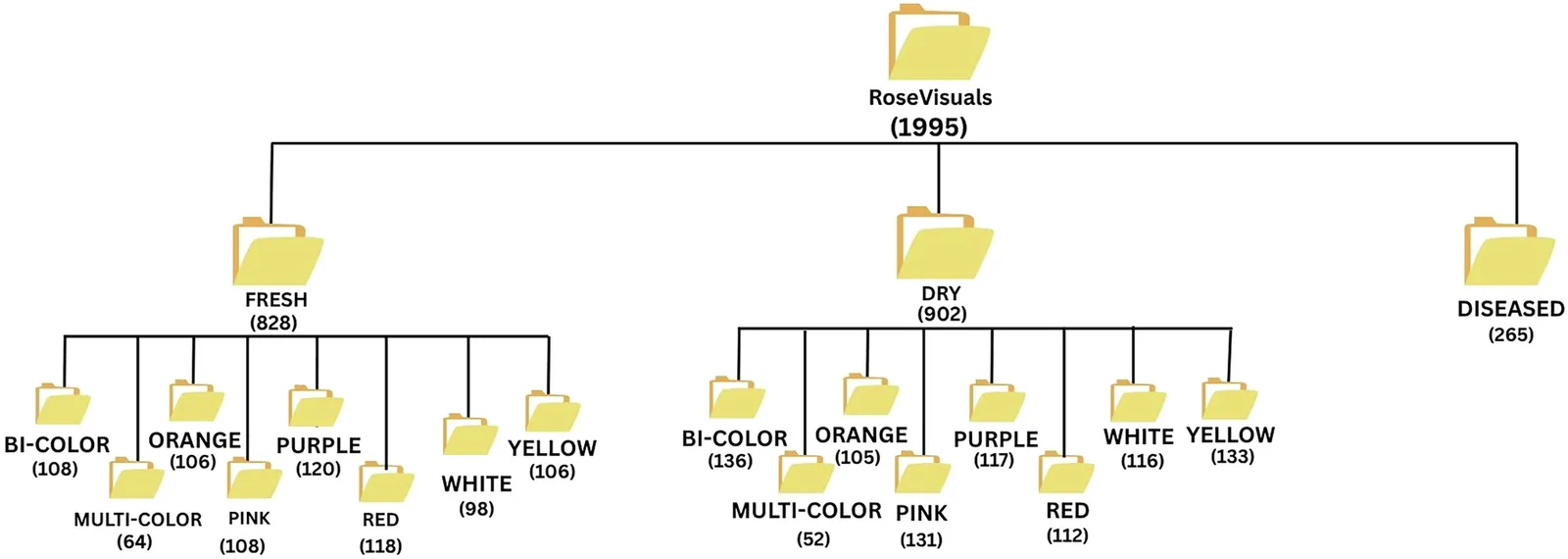
Data Organization.

The dataset comprises 1,995 high-quality images of Dutch rose petals, categorized on the basis of health status and color characteristics^7^. The dataset is organized at the top level based on petal health conditions—fresh, dry, and diseased—where the fresh and dry category is further classified into different color varieties such as bi-color, multi-color, orange, pink, purple, red, white, and yellow. The dataset includes petals collected from multiple plants across different farms and collection days. While specific cultivar names were not individually recorded, the dataset represents commonly cultivated Dutch rose varieties grown for commercial purposes. The class distribution reflects natural farm availability rather than enforced balance, providing a realistic representation of real-world conditions. Figure 4 depicts the hierarchical organization of the RoseVisuals dataset.

All images are captured on Samsung S21 Ultra using Pro camera mode with built-in flash with a consistent resolution of 3000 × 4000 pixels against a simple, neutral background to minimize external factors that could affect accurate classification. Some of the Images, which were captured in an the auto-landscape orientations were then manually switched into portrait to give more consistency. and without overprinting or replication of images, retaining the original 3000 × 4000 pixels. format. There were no augmentations. The pictures are in the form of JPG. Each image in the dataset is labeled distinctively with unique identifier. Petal health is classified as follows: Fresh petals show high turgidity, vibrant color, and smooth texture, reflecting their fresh and tender harvest state. Dry petals were sundried for three days with 5-6 hours of daily exposure at 25–30 °C to complete the drying process. Diseased petals exhibit noticeable harm caused. by such infections as black spot, rust, and botrytis.

Table 2. Data statistics. Besides the image files, the repository additionally includes annotation files in CSV format. These annotations record image metadata with image-level data such as petal health condition, color type and location of collection. To facilitate the reproducibility and external use of the instruments, the repository has a comprehensive README file that outlines the structure of the folders, definitions of the classes, and the conventions that are used in naming the files and the number of samples in each category. The labels of the disease in the dataset are made only on basis of visual appearance. Although some visible symptoms might be observed, including the spotting, discoloration, or tissue degradation, there is no particular type of disease detected or clinically proven during fresh petal gathering.

**Table 2:** Data Statistics of the dataset.

COLOR VARIETY	TOTAL IMAGES	FRESH	DRY	DISEASED
	244	108	136	0
	116	64	52	0
	211	106	105	0
	239	108	131	0
	237	120	117	0
	230	118	112	0
WHITE	214	98	116	0
	239	106	133	0
TOTAL COLOR VARIETIES	1730			
TOTAL IMAGES	1995	828	902	265 (Diseased not categorized by color)

Depicts some of the quantitative statistics regarding the data collected by the RoseVisuals dataset. The Dutch rose petal images are categorized, and some quantification with regard to the distribution of 1995 high-resolution images of rose petals with different color varieties and the health condition, whether fresh or diseased. There are altogether 1,730 images, spread across eight different color varieties, observed in the fresh as well as dry states, to varying degrees. In the disease condition, there are altogether 265 images, which are primarily classified under irregularities in coloration and significant visual interference.

The RoseVisuals dataset along with required documentation is accessible publicly at Mendeley Data and available through the. direct https://data.mendeley.com/datasets/f44jwtbfjg/4. The dataset is referred to as. the 10.17632/f44jwtbfjg.4 and is named RoseVisuals in the repository^7^.

## Technical Validation

In order to achieve technical quality, consistency, and reliability, the RoseVisuals dataset underwent some technical validation processes. Significant validation measures have been established. throughout the stages of research. Borderline cases with minor discoloration without clear pathological indicators were excluded from the diseased category and retained under fresh or dry classes as appropriate based on visual apperance. Only petals that show clear signs of damage or infection patterns were labeled as diseased.

### Image acquisition protocol and quality control

All the Images of the dataset, namely RoseVisuals, have been collected systematically via a Samsung SMG998B smartphone model, thereby reducing potential variability resulting from camera equipment differences. Subsequent to this, all Images have been subjected to entire manual scrutiny, with the intention of removing Images that display any form of technological flaw, such as motion blur, improper focusing, under- and over-exposure, and high levels of brightness, respectively. All Images have been scrutinized to ensure that they do not display any kind of artificial alteration, filters, image enhancements, and static augmentation, respectively, with the intention of reducing variability and maintaining raw dataset authenticity, respectively. Double verification of all Images, concerning their resultant labeling, was conducted by all of the authors of the manuscript who took part in the creation of this dataset, with every author being sufficiently trained and knowledgeable in computer sciences, respectively. Consequently, considering that there was no expert plant pathologist verification, relatively conservative labeling was conducted Unique IDs made auditing dataset and reproducing experimental findings from the RoseVisuals.

### Duplicates detection and removal

A duplicate detection pipeline that minimizes redundancy and guarantees the uniqueness of the dataset was created. This involved using the imagehash library. This technique entailed generating a perceptual hash for all images. Then, a criterion of Hamming distance ≥ 5 was used to recognize images that are visually similar or near-duplicate. This technique exploits characteristics of images that are likely to go undetected when using alternative techniques. The system managed to detect duplicates even under mild orientation or color changes. This helped to identify duplicate or near-duplicate images, thereby optimizing the variety of this dataset to minimize any possible bias that would occur if duplicates were used.

### Metadata extraction and verification

The implementation of a metadata extraction technique using Python illustrated how metadata compilation from images embedded deep within their respective files and can be achieved very efficiently. The metadata compilation involved obtaining information ranging from image dimensions and resolution to timestamp and camera model. Compilation of this metadata formed a CSV file that helped to validate that all images were indeed captured by Samsung devices’ brand model SM-G998B.Confirming uniform resolution and image dimensions across the dataset to prevent downstream issues related to variance in image scale. Detecting any indications of artificial post-processing or image alteration by analyzing photographic metadata and ensuring no discrepancies were present.

Verifying image dimensions and resolution are uniform across the dataset prevents issues stemming from variance in scale effectively.

## Usage Notes

Despite the application of rose petals in cosmetics production, food industries, and perfume industries, there is a lack of petal-faced datasets. Most of the floral datasets available today showcase entire flowers, which makes them less effective for tasks that require detailed, petal-level analysis. While extensive research has been conducted on the Iris flower, rose petals haven’t received the same attention, especially in the context of machine learning applications^8^. Although the dataset size may be modest for training large end-to-end deep learning models, RoseVisuals is intended primarily as a high-quality, fine-grained benchmark for transfer learning, feature extraction, and comparative evaluation rather than large-scale model training from scratch. Petals assessment, especially for commercial purposes alongside disease detection and variety of colors, remains absent from general flower databases despite their assessment value. The Flower Recognition flower dataset on Kaggle features roses; however, it focuses on species, not on qualitative characteristics of flowers, especially for industrial purposes. To address this gap, RoseVisuals serves as an organized resource emphasizing the commercial and visual characteristics of rose petals. The dataset supports the development of predictive models that enhance supply chain management by monitoring deterioration patterns across various color categories^9^. Table 3 presents a comparison between the existing datasets and the proposed dataset-RoseVisuals.

**Table 3:** Comparison Table.

Dataset	Total Samples	Color Varieties	Petal Health Considerations	Focus on Main Petal
Rose Flowers Dataset^10^	72	Not Specified	Not Considered	Absent
Rose Leaves Disease^11^	917	Not Specified	Not Considered	Absent
Flower Recognition^12^	4.242	Multiple (Daisy, Dandelion, Rose, Sunflower, Tulip)	Not Considered	Absent
RoseVisuals: A Multi-Class Dutch Rose Petal Images Dataset for Automated Health and Pigmentation Classification via Deep Learning^7^	1.995	Multiple (red, white, pink, purple, orange, yellow, bicolor and multicolor)	Considered. Fresh, Dry and Diseased.	Yes

Depicts a Comparative analysis of RoseVisuals against existing floral image datasets. This table highlights the differences in scope, structure, and level of detail among four datasets: Rose Flowers, Rose Leaves Disease, Flower Recognition, and the proposed RoseVisuals dataset. The comparison includes total sample count, color variety coverage, consideration of petal health, and whether the dataset specifically focuses on the petal level.

## Data Availability

The RoseVisuals dataset is publicly available on Mendeley Data at Direct URL to data: https://data.mendeley.com/datasets/f44jwtbfjg/5. Data Identification Number: 10.17632/f44jwtbfjg.5. Repository Name: RoseVisuals.

## Code availability

Scripts for validating datasets and extracting metadata were implemented using Python. Custom Scripts and Utilities guarantee technical quality and transparency of the RoseVisuals dataset with bespoke tools ensuring consistency of the RoseVisuals dataset. Imagehash and PIL libraries computed perceptual hashes of each image quite effectively. The RoseVisuals codebase, comprising all validation scripts, is publicly available on GitHub Repository at https://github.com/Arya-S14/RoseVisuals-Validation-Doc.

Custom Scripts and Utilities comprises.

1. Duplicate Image Detection - duplicatechecker.py.

2. Duplicate Image Detection Output file - duplicates_report.txt.

3. Images Metadata Extraction - metadata_extraction.py.

4. Images Metadata - images_metadata.csv.
